# Supplementary material for: Asymmetric Synthesis of Quaternary Hydantoins via a Palladium-Catalyzed Aza-Heck Cyclization
Source: J Am Chem Soc. 2025 Nov 14;147(49):44692–8. doi: 10.1021/jacs.5c16022 (PMC12703750; doi:10.1021/jacs.5c16022)
Supplement: Supplementary file 2 [file ja5c16022_si_002.zip › All NMR FID Files/S24/S24_AllNMR/2/pdata/1/email_EM03-050_2_1.pdf]

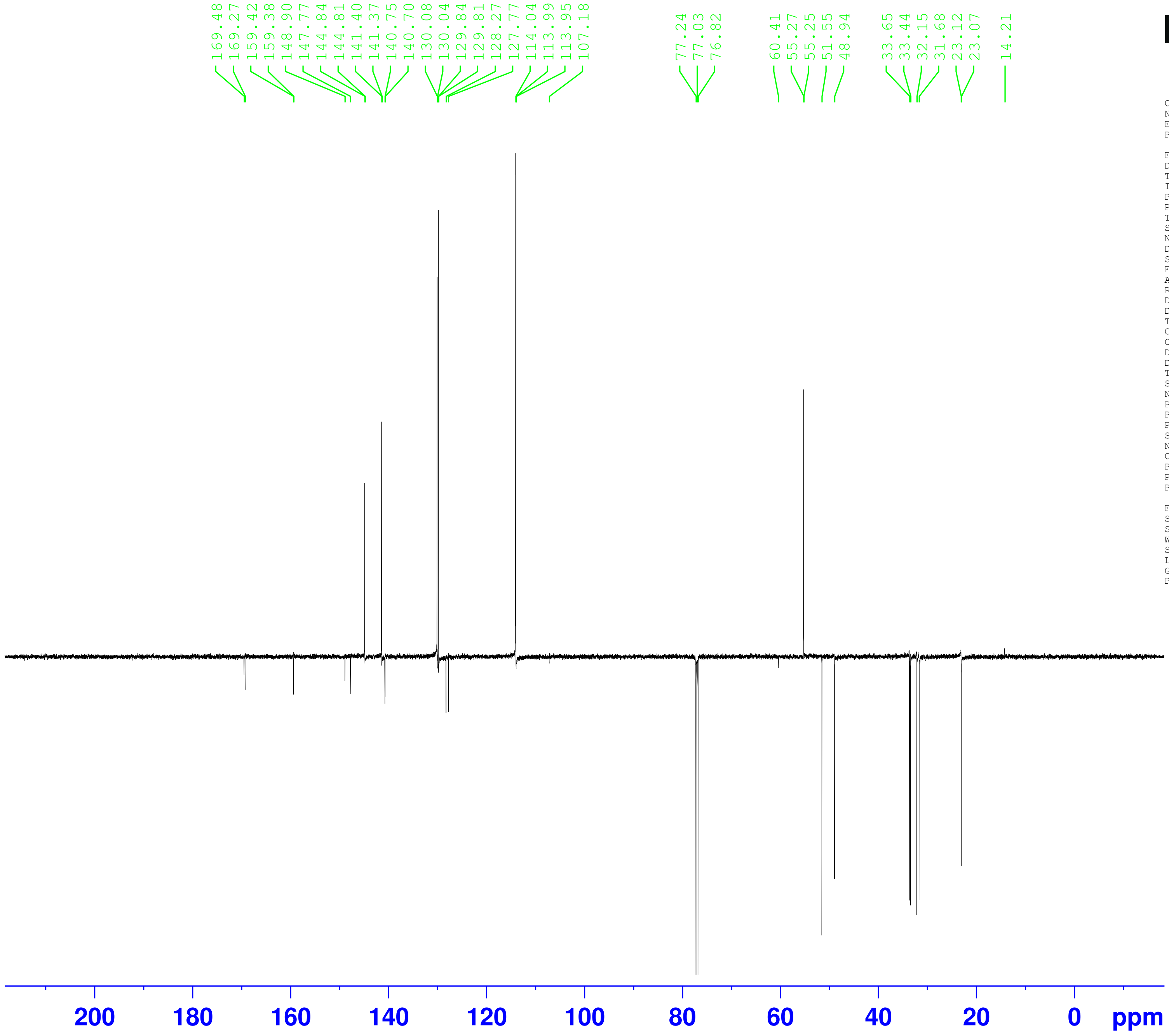

BRUKER

```
Current Data Parameters
NAME              EM03-050
EXPNO                    2
PROCNO                   1

F2 - Acquisition Parameters
Date_             20220827
Time                 16.18 h
INSTRUM   Avance Neo 600 at University of Delaware
PROBHD   Z185170_0001 (
PULPROG               jmod
TD                   65536
SOLVENT              CDCl3
NS                     256
DS                       4
SWH              35714.285 Hz
FIDRES            1.089913 Hz
AQ               0.9175040 sec
RG                     101
DW                  14.000 usec
DE                   19.25 usec
TE                   298.0 K
CNST2       145.0000000
CNST11        1.0000000
D1           2.00000000 sec
D20          0.00689655 sec
TD0                      1
SFO1       150.9178988 MHz
NUC1                   13C
P1                   12.00 usec
P2                   24.00 usec
PLW1       100.76999664 W
SFO2       600.1324005 MHz
NUC2                    1H
CPDPRG[2           waltz65
PCPD2                80.00 usec
PLW2          6.89699984 W
PLW12         0.06921953 W

F2 - Processing parameters
SI                   32768
SF         150.9028085 MHz
WDW                     EM
SSB                      0
LB                    1.00 Hz
GB                       0
PC                    1.40
```
